# Do women who consult with naturopaths or herbalists have a healthy lifestyle?: a secondary analysis of the Australian longitudinal study on women’s health

**DOI:** 10.1186/s12906-020-03153-6

**Published:** 2020-11-18

**Authors:** Amie Steel, Stefania Tiveron, Rebecca Reid, Jon Wardle, Holger Cramer, Jon Adams, David Sibbritt, Romy Lauche

**Affiliations:** 1 Australian Research Centre in Complementary and Integrative Medicine (ARCCIM), Faculty of Health, University of Technology Sydney, Level 8, Bldg 10, 235-253 Jones St, Ultimo, NSW, Sydney Australia; 2 Sick Kitty Naturopathic Clinic, Ontario, Canada; 3 Department of Internal and Integrative Medicine, Evang. Kliniken Essen-Mitte, Faculty of Medicine, University of Duisburg-Essen, 45276 Essen, Germany

**Keywords:** Health behaviours, Health promotion, Naturopathy, Illicit drugs, Dietary practices

## Abstract

**Background:**

Australians report consulting with a naturopaths or herbalists to improve their wellbeing, yet little is known about the associations between these consultations and the patients’ health behaviours. This cross-sectional study aimed to examine the relationship between health behaviour and consultations with naturopaths or herbalists in three age cohorts of Australian women.

**Methods:**

Women aged 19–25 years, 31–36 years, and 62–67 years from the Australian Longitudinal Study on Women’s Health (ALSWH) were surveyed regarding smoking, alcohol or drug use, physical activity and dietary behaviour; and whether they consulted with naturopath/herbalists in the last 12 months. Associations were analysed using multivariable logistic regression.

**Results:**

A total of 9151 (19–25 years), 8200 (31–36 years) and 11,344 (62–67 years) women were included in the analysis. Between 7.3 and 11.9% of women reported to have consulted with naturopaths/herbalists in the last 12 months. Women of all cohorts consulting with naturopath/herbalist were less likely to smoke (19-25 yrs.: Odds Ratio [OR] 0.61; 31–36 years: OR 0.58; 62–67 years: OR 0.29), more likely to report at least moderate levels of physical activity (19-25 yrs.: OR 1.41; 31–36 years: OR 1.34; 62–67 years: OR 1.34), and the use of vegetarian diets(19-25 yrs.: OR 1.40; 31–36 years: OR 1.77; 62–67 years: OR 2.28), compared to women not consulting with naturopaths/herbalists. Women consulting with naturopaths/herbalists however were also more likely to have used marijuana (19-25 yrs.: OR 1.18; 31–36 years: OR 1.42), or illicit drugs in the last 12 months (19-25 yrs.: OR 1.24; 31–36 years: OR 1.40).

**Conclusions:**

Consultations with a naturopath or herbalist are associated with positive health behaviours that are protective of internationally important non-communicable diseases. Psychoactive drug use is also reported among women visiting a naturopath or herbalist. Further research is needed to understand the role naturopaths play in advising patients with regards to health and non-healthy behaviours.

## Background

Naturopathy is a traditional system of medicine which originates from Europe but is now practiced in over 80 countries across every world region [1]. The practice of naturopathy is defined by core principles and underpinned by philosophies which emphasise preventive medicine and patient education [2]. Naturopathic training varies based on the regulatory framework of the country it is practiced, but most commonly includes diet and lifestyle counselling followed by herbal medicine and clinical nutrition [3]. Naturopathy has also been argued to align with public health principles [4] and naturopaths in some countries are considered to be trained as primary care practitioners [5]. Naturopaths also treat diverse populations with varied health conditions and across all age groups [6]. Their consultations may include discussion of numerous health topics [6], and a strong focus on health behaviour change [7]. Individuals consulting with a naturopath have described positive experiences of practitioner empathy and qualities of patient-centred care including empowerment and support for lifestyle and behaviour change [8, 9]. In Australia, the naturopathic profession has been closely tied to Western herbal medicine, with the peak professional and regulatory bodies for naturopathy also representing herbalists [10, 11]. Approximately 6.2% of Australians have consulted with a naturopath and 3.8% have visited a herbalist in the previous 12 months and over half of those are consulting with a naturopath or herbalist to improve their well-being [12].

A number of factors characterise individuals that use complementary medicine (CM) services [13, 14] and may impact on the overall outcomes of care provided by a naturopath or Western herbalist [15]. For example, psychosocial factors such as spirituality and holistic belief systems are linked to complementary medicine (CM) use [14, 16, 17]. A mind-set which values a holistic approach is also compatible with having a higher internal health locus of control - a health belief that the outcome of one’s health seeking is related to their own actions [18] – and as such a holistic belief system may influence the uptake and maintenance of self-care health behaviours. Equally, research has shown that health-aware behaviours and dissatisfaction with conventional medicine are strong predictors of CM health services [13].

In the context of the global burden of disease resulting from chronic illness, factors that influence both positive and risky health behaviours warrant close attention. Dietary choices, physical activity, alcohol consumption, and tobacco smoking impact significantly on the risk of non-communicable diseases [19–25] that are not only of international importance [26], but also seen as a priority at a national level by countries like Australia [27]. A strong focus of strategies aimed at addressing these diseases is to improve the delivery of primary health care and improve protective health behaviours in the community [27], including improving the delivery of integrated health care, diagnosis and treatment.

Despite the current knowledge regarding CM use and health behaviours, little is known about the health behaviours associated with consultations with specific health profession such as naturopaths and herbalists. Thus, the aim of this study was to analyse associations between health behaviours and visits with a naturopath or Western herbalist in a large nationally representative sample of Australian women.

## Methods

This study presents analysis of data from the Australian Longitudinal Study on Women’s Health (ALSWH) [28]. The ALSWH is a nationally representative study that was designed to assess health and wellbeing in Australian women [29]. The study was established in 1996 with three different age cohorts (1921–1926, 1946–1951, and 1973–1978 cohort) randomly selected from the national Medicare database [28]. In 2012 a new cohort was also introduced (1989–1995 cohort).

This study presents analysis of data from 9151 women from the 1946–1951 cohort (Survey 7, 2013; respondents aged 62–67 years), 8200 women from the 1973–1978 cohort (Survey 5, 2009; respondents aged 31–36 years), and 11,345 women from the 1989–1995 cohort (Survey 2, 2014; respondents aged 19–25 years). These datasets were chosen for the analysis as they provided the largest number of outcomes relevant to our research question.

### Consultation with naturopaths/herbalists

The women were asked if they had consulted with a naturopath/herbalist in the past 12 months on a yes/no basis.

### Smoking

Survey items were included that enquired about whether women were smoking cigarettes or tobacco products, or whether they had smoked in the past. Women who reported to currently smoke were asked to provide details about the frequency and quantity.

### Alcohol use

A series of survey items reporting frequency and amount of alcohol consumption were also included in the analysis.

### Marijuana use

Women in the 1973–1978, as well as the 1989–1995 cohorts were asked whether they had used marijuana (Cannabis, hash, grass, dope, pot, yandi) for non-medicinal purposes. Their response options were: *never*, *more than 12 months ago*, *in the last 12 months*.

### Illicit drug use

Survey items that captured whether women they had used illicit drugs (Amphetamines, LSD, natural hallucinogens, tranquilizers, cocaine, ecstasy, inhalants, heroin or barbiturates) for non-medicinal purposes were used from the surveys admininstered to the 1973–1978 and 1989–1989 cohorts. Respondents could choose between *never*, *more than 12 months ago*, *in the last 12 months*.

### Physical activity

A series of survey items examined physical activity by determining the type of activity (walking, moderate, vigorous activities), and frequency/duration of the activity within the past week.

### Dietary behaviour

The Dietary Questionnaire for Epidemiological Studies Version 2, a 101-item food frequency questionnaire (FFQ) [30, 31] was included in the surveys used for this analysis and provided the data for examining respondents’ dietary behaviour.

### Statistical analyses

The data was initially recoded to enable the analysis needed to answer the research question. A binary variable was generated for smoking in which those who smoke regularly (on a weekly/daily basis]) were grouped separately to those who do not (including non-smokers, and occasional smokers). Alcohol use was recoded according to the National Health and Medical Research Council (NHMRC) classification [32], and recoded for the comparisons of those with high risk drinking behaviour (including risky drinkers, and high risk drinkers) vs. those without (including non-drinkers, low risk drinkers). Variables were created that compared women reporting Marijuana use in the past 12 months with those who did not. A similar variable was generated for illicit drugs use. Responses to the physical activity survey items were converted to metabolic equivalents (MET), with one MET equivalent to 3.5 mL of oxygen uptake per kg per minute. Based on their MET level women were classified as sedentary (0–33.2), low active (33.3–499), moderate (500–999) or high active (≥1000). The recoding followed the guidelines from the Active Australia physical activity survey [29]. Based on the survey items reporting the the frequencies of food items consumed as part of respondents’ usual eating habits in the past 12 months, women were classified as following a full-time vegetarian diet (no meat, no poultry), a vegan diet (no animal products including meat, poultry, eggs, dairy products of any kind), or an other diet. For the analysis, those following a vegetarian or vegan diet were compared to those who do not.

Analyses were conducted separately for the three age cohorts. Separate logistic regressions were conducted to determine whether smoking, alcohol, marijuana, or illicit drug use, physical activity and vegetarian diet were associated with the consulting with naturopaths/herbalists. Adjusted odds ratios with 95% confidence intervals were computed for the independent variable. Analyses were adjusted for socio-demographic characteristics and confounding variables (socioeconomic status including marital status, education, income, area of residence, body mass index, and self-reported doctor diagnosed depression). Statistical significance was set at *p* < 0.05. All statistical analyses were performed using IBM SPSS® software (IBM SPSS Statistics for Windows, release 22.0. Armonk, NY: IBM Corp.).

## Results

The prevalence of consulting with naturopaths/herbalists in each cohort can be found in Table 1. Highest prevalence of naturopath/herbalist consultations can be found in the 1973–1978 cohort (11.9%), followed by the 1989–1995 cohort (8.6%) and the 1946–1951 cohort (7.3%). Alcohol was consumed by the vast majority of women, however only a minority showed high risk or risky drinking behaviour, with lower prevalence in the younger cohort. A substantial proportion of women, especially in the 1989–1995 cohort, reported having used marijuana in the past 12 months (28.8%) compared to only 9.2% in the 1973–1978 cohort. Most women in the cohorts were physically active with the oldest and youngest cohorts showing highest prevalence for at least moderate physical activity levels. Vegetarian dietary choices were however only reported by a minority of the older cohorts, and only in the 1989–1995 cohort was a substantial prevalence found.

**Table 1:** Frequency of health behaviours in 9151 Australian women aged 62–67 years (1946–1951 Cohort), 8200 Australian women aged 31–36 years (1973–1978 Cohort), and 11,344 Australian women aged 19–25 years (1989–1995 Cohort), in % of all women in the respective cohort. ^NA^ = Not assessed

Variable	1946–1951 Cohort	1973–1978 Cohort	1989–1995 Cohort
Consulting with naturopath/herbalist	7.3	11.9	8.6
Smoking (≥weekly)	4.8	12.1	3.8
Alcohol use (high risk/risky drinking)	6.0	4.4	2.9
Marijuana use (last 12 months)	NA	9.2	28.8
Illicit drug use (last 12 months)	NA	6.1	16.5
Physically active (≥moderate)	61.5	49.8	68.8
Vegetarian/vegan diet	2.0	3.8	8.7

Table 2 shows the associations between health behaviours and naturopath/herbalist consultations. Women in all three cohorts were less likely to smoke (Odds Ratio OR: 0.29 [0.15–0.56], 0.58 [0.44–0.75], 0.61 [0.39–0.95], from the oldest to youngest age groups respectively) when they consulted with naturopaths/herbalist, compared to those who did not. Women consulting with naturopaths/herbalists were more likely to report at least moderate levels of physical activity (OR: 1.34 [1.10–1.62; 1.16–1.55], 1.41 [1.20–1.65], from the oldest to youngest age groups respectively), and report vegetarian/vegan dietary practices (OR: 1.40 [1.13–1.74], 1.77 [1.30–2.41], 2.28 [1.47–3.52], from the oldest to youngest age groups respectively), compared to women not consulting with naturopaths/herbalists. On the other hand, compared to women not consulting with naturopaths/herbalists, women who did were also more likely to report higher likelihoods of marijuana use (OR: 1.18 [1.02–1.37], 1.42 [1.14–1.78], from the oldest to youngest age groups respectively), or illicit drug use in the last 12 months (OR: 1.24 {1.05–1.48], 1.40 [1.07–1.83], from the oldest to youngest age groups respectively). The patterns of associations were similar across all cohorts of women.

**Table 2:** Output from the logistic regression models showing the association between consulting with a naturopath/herbalist and several health behaviour categories, in 9151 Australian women aged 62–67 years (1946–1951 Cohort), 8200 Australian women aged 31–36 years (1973–1978 Cohort), and 11,344 Australian women aged 19–25 years (1989–1995 Cohort). OR: Odds Ratio; CI: Confidence interval. Confounder included in the analysis: Body mass index, depression, marital status, income management, area, education

Health behaviours	Likelihood of health behaviours among women consulting with a naturopath/herbalist compared to women who do not consult with a naturopath/herbalist					
	1946–1951 Cohort		1973–1978 Cohort		1989–1995 Cohort	
	Odds ratio (95% CI)	*p*	Odds ratio (95% CI)	*p*	Odds ratio (95% CI)	*p*
Smoking						
*Not smoking or irregular smoking (≤weekly)*	ref	*–*	ref		ref	
*Smoking regularly (≥weekly)*	0.29 (0.15, 0.56)	*< 0.001*	0.58 (0.44, 0.75)	*< 0.001*	0.61 (0.39, 0.95)	0.028
Alcohol use						
*No risk/low risk*						
*High risk/risky*	0.74 (0.49, 1.11)	0.144	0.77 (0.54, 1.11)	0.163	0.72 (0.45, 1.14)	0.162
Marijuana use						
*Did not use in the last 12 months*	Ref		Ref		Ref	
*Used in the last 12 months*			1.42 (1.14, 1.78)	0.002	1.18 (1.02, 1.37)	0.029
Illicit drug use						
*Did not use in the last 12 months*	–	–	Ref		Ref	
*Used in the last 12 months*	–	–	1.40 (1.07, 1.83)	0.013	1.24 (1.05, 1.48)	0.014
Physical activity						
*Low/sedentary*	ref		ref		ref	
*Moderate*	1.34 (1.10, 1.62)	0.003	1.34 (1.16, 1.55)	< 0.001	1.41 (1.20, 1.65)	< 0.001
Vegetarian diet						
*Not vegetarian/vegan*	ref		ref		ref	
*Vegetarian/vegan*	2.28 (1.47, 3.52)	< 0.001	1.77 (1.30, 2.41)	< 0.001	1.40 (1.13, 1.74)	0.002

## Discussion

This article represents the first report from a nationally representative sample of women which examines the associations between consulting with a naturopath or herbalist and their health behaviours. The results suggest that women from all three cohorts exhibit differences in lifestyle behaviours associated with consultations with a naturopath or herbalist. Naturopathic principles include a strong focus on educating individuals to modify their health behaviours to improve their health status [2]. Patients consulting with a naturopath have also reported empowerment as a feature of the naturopathic consultation [8, 9] while clinical research has highlighted the emphasis placed on modifying dietary and lifestyle behaviours within the naturopathic system of healing [15]. Equally, individuals that choose to consult with a naturopath may be predisposed to specific health behaviours due to their own individual holistic personal belief systems [14, 16, 17]. It is unknown whether consulting with a naturopath/herbalist empowers women to adopt the health behaviours discussed in this article, or if personal characteristics and psychosocial factors driving these health behaviours also predict intention and openness to seek naturopathic/herbalist care. Given that health aware behaviours are subject to interpretation based on one’s values, beliefs and philosophical orientations towards health, consulting with a naturopath/herbalist may align with a similar belief system. However, the directionality of the relationship between health behaviors and consultation with a naturopath can not be confirmed due to the cross-sectional nature of the study.

Women consulting with naturopaths/herbalists were more likely to report engaging in positive health behaviours such as not smoking, at least moderate levels of physical activity, and following a vegetarian or vegan diet. These health behaviours are known to reduce the risk of diseases that contribute a significant burden on contemporary health systems such as cardiovascular disease [19–21], metabolic syndrome [22, 23], cancer [19, 21], and mental illness [24, 25]. Within the context of cardiovascular disease, a 2011 finding that the risk attributable to coronary heart disease from smoking is greater in women compared to men [20] becomes particularly important given Australian women are more likely than men to consult with a naturopath or herbalist [12]. In this context, if naturopaths or herbalists are engaged in lifestyle counselling which encourages smoking cessation [6], then these health professions may have a potential role in addressing smoking prevalence in female populations. Alternatively, if the decision to engage in these positive health behaviours is made prior to consultation with a naturopath or herbalist then the users’ view of naturopathy and herbal medicine within their wider approach to health warrants closer investigation. US research proposes different characteristics among CM users based on whether they access CM for health promotion or treatment [33]. With this in mind, it is possible that some individuals may follow these positive health behaviours before they choose to consult with a naturopath or herbalists, while others may engage in such behaviours as a result of the interaction with or advice from the naturopath or herbalist.

Alcohol was consumed by most of the women who consulted with a naturopath in our study, however only a minority showed high risk or risky drinking behaviour particularly in the younger cohort; and alcohol consumption was not associated with the (non-) consultation of naturopaths/herbalists. Previous research in the US has found CM users also have a higher incidence of consuming alcohol daily but not meeting the classification of heavy drinking [34]. Other research elsewhere also found alcohol consumption was not a significant predictor of CM use [35, 36]. The health impacts of alcohol consumption have received increased interest among public health researchers in recent years in light of conflicting findings regarding the potential risk and benefits associated with ‘moderate’ compared with ‘heavy’ alcohol use or abstinence [37]. While some groups argue that the beneficial effects attributed to alcohol consumption may differ based on the type of alcohol consumed [38], epidemiological meta-analyses indicate no positive health benefits to all-cause mortality for moderate alcohol use [39]. Within the context of naturopathy and herbal medicine users, while contemporary research indicates nine out of ten naturopaths in Australia ‘sometimes’ or ‘often’ discuss the alcohol use with their patients [6], little is known about the specific advice regarding alcohol consumption given to patients by clinicians irrespective of the changing consensus towards alcohol’s health effects.

This study also reports a higher prevalence of marijuana use or illicit drug use in women who consult with naturopaths/herbalists, compared to those who do not. The apparent contrast between the prevalence of other positive health behaviours and the use of psychoactive drugs may be explained by previously reported perceptions of self-reported health benefits among drug users [40]. Over 80% of users also reported having had a ‘spiritual experience’ from a psychoactive drug [40]. Given the potential relationship between CM use and spiritual beliefs [41], a further exploration of the higher illicit drug use among users of naturopaths and herbalists may provide new insights into the role of psychoactive substances in spiritual practice for some individuals. An alternative explanation for the pattern of illicit drug use among users of naturopath and herbalist services is the potential for both behaviours (drug use and naturopath/herbalist consultations) to be part of an individual’s counterculture lifestyle [42, 43]. Previous research also suggests the personality trait “openness to new experience” as a predictor of CM use in general, but specifically with the decision to initially try and explore CM [44], and this characteristic may also contribute to a decision to use marijuana [45]. Furthermore, some illicit drugs may be associated with perceived benefits that appeal to healthy individuals looking to enhance academic and work performance, especially stimulant medications such as amphetamine [46]. Irrespective of the reason for illicit drug use, this research emphasises the need for naturopaths and herbalists to be appropriately trained to enquire about illicit drug use and counsel behaviour change among individuals using illicit drugs. While many Australian naturopaths not only discuss substance use with their patients, approximately one third report ‘sometimes’ or ‘often’ treating drug and alcohol addiction [6]. However, as this is the first time to our knowledge that substance use behaviours among naturopathy or herbal medicine users has been reported, further research is needed to understand the dynamic between these health behaviours and this sub-population.

### Limitations

The findings from this study should be considered in light of its limitations: naturopath/herbalist consultations were assessed as one item; therefore, it cannot be identified which practitioner was consulted. Furthermore, the data are based on self-reports and women may not have recollected all health behaviour details correctly, and a social desirability bias cannot be ruled out. Despite these limitations, the ALSWH is a comprehensive and well-respected source for epidemiological data and the large number of participants as well as the inclusion of the most important confounders within the regression models provides strength to the analyses reported here.

## Conclusions

There is a difference in health behaviours among women that consult with a naturopath or herbalist compared to women that do not, and these differences have important implications in public health policy and primary health service delivery. Naturopaths and herbalists may have an influential role in supporting positive health behaviours and counseling behaviour change among individuals identified as engaging in risky behaviours. The gaze of health research and policy needs to widen to encompass health professions such as naturopathy and herbal medicine and capitalise on their potential contribution to population health.

## Data Availability

The data that support the findings of this study are available from Women’s Health Australia but restrictions apply to the availability of these data, which were used under license for the current study, and so are not publicly available. Data are however available from the authors upon reasonable request and with permission of Women’s Health Australia.

## Abbreviations

ALSWH: Australian Longitudinal Study on Women’s Health
CM: Complementary medicine
DOHA: Department of Health and Ageing
FFQ: Food frequency questionnaire
MET: Metabolic equivalents
NHMRC: National Health and Medical Research Council
OR: Odds ratio
US: United States
